# Neuropsychiatric- and cognitive post-acute sequelae of SARS-CoV-2 infection – evidence from K18-hACE C57BL/6 J mice

**DOI:** 10.1093/ijnp/pyaf072

**Published:** 2025-09-30

**Authors:** Marco Maria Santi, Eleonora Genovese, Thor Mertz Schou, Matheus da Silva, Sophie Erhardt, Lilly Schwieler, Jacob Ahlberg Weidenfors, Giorgia Marino, Søren Riis Paludan, Samia Joca, Gregers Wegener, Line Reinert, Cecilie Bay-Richter

**Affiliations:** Translational Neuropsychiatry Unit, Department of Clinical Medicine, Aarhus University, Aarhus N 8200, Denmark; Department of Life Sciences, University of Trieste, Trieste, Italy; Translational Neuropsychiatry Unit, Department of Clinical Medicine, Aarhus University, Aarhus N 8200, Denmark; Department of Life Sciences, University of Trieste, Trieste, Italy; Translational Neuropsychiatry Unit, Department of Clinical Medicine, Aarhus University, Aarhus N 8200, Denmark; Translational Neuropsychiatry Unit, Department of Clinical Medicine, Aarhus University, Aarhus N 8200, Denmark; Department of Physiology and Pharmacology, Karolinska Institute, Stockholm, Sweden; Department of Physiology and Pharmacology, Karolinska Institute, Stockholm, Sweden; Department of Physiology and Pharmacology, Karolinska Institute, Stockholm, Sweden; Department of Biomedicine, Aarhus University, Aarhus, Denmark; Center for Immunology of Viral Infections, Aarhus University, Aarhus, Denmark; Department of Biomedicine, Aarhus University, Aarhus, Denmark; Center for Immunology of Viral Infections, Aarhus University, Aarhus, Denmark; Department of Biomedicine, Aarhus University, Aarhus, Denmark; Translational Neuropsychiatry Unit, Department of Clinical Medicine, Aarhus University, Aarhus N 8200, Denmark; Department of Biomedicine, Aarhus University, Aarhus, Denmark; Center for Immunology of Viral Infections, Aarhus University, Aarhus, Denmark; Translational Neuropsychiatry Unit, Department of Clinical Medicine, Aarhus University, Aarhus N 8200, Denmark

**Keywords:** post-acute sequelae of SARS-CoV-2 infection, cytokines, kynurenine pathway, microbiome, cognition

## Abstract

**Background:**

Survivors of COVID-19 frequently report psychiatric and cognitive sequelae. The origin of such sequelae has not been determined, as it has been a challenge to resolve whether these symptoms have a viral origin or are related to the contextual stressors associated with the pandemic. Using a mouse model of post-acute sequelae of SARS-CoV-2 infection (PASC), we examined neurobiological mechanisms underlying these effects without the confounding influence of contextual factors.

**Results:**

SARS-CoV-2 infection induced cognitive, but not anxiety- or depression-like, behavioral deficits. Cognitive impairments correlated with severity of the acute disease. Infected mice showed significant alterations in brain cytokine levels, as well as in kynurenine pathway (KP) metabolites, both of which were associated with acute disease severity. Microbiome taxonomic profiling revealed group-specific differences, indicating that certain bacterial species may contribute to PASC development.

**Conclusions:**

Our findings suggest that SARS-CoV-2 infection causes cognitive deficits in PASC, modulated by acute disease severity, while anxiety- and depression-like behaviors appear unrelated to the viral infection itself. This supports the idea that such psychiatric symptoms may stem from pandemic-related stressors rather than infection. Altered cytokine signaling and KP metabolism may play key roles in the pathophysiology of PASC, identifying potential biomarkers and therapeutic targets.

## INTRODUCTION

COVID-19 has a multiorgan pathology.^1^ In addition to the well-described respiratory and cardiovascular symptoms, more than one-third of the infected patients develop neurological symptoms in the acute phase,^2^ and many show brain abnormalities.^3^ Several studies have reported a high incidence of acute psychiatric symptoms in COVID-19 patients.^4^ Data on long-term effects of SARS-CoV-2 infection describe a multitude of symptoms, including breathlessness, chest pains, and fatigue. Intriguingly, psychiatric symptoms may also persist long after recovery from the initial infection.^5^

The causes of the psychiatric sequelae are not clear, since it has been a challenge to determine if they are triggered by the virus or the contextual stressors associated with the pandemic, such as lockdowns and social distancing. A recent study reported that one-third of the subjects suffering from post-acute sequelae of SARS-CoV-2 infection (PASC) did not have antibodies against SARS-CoV-2 nucleocapsid, indicating that they had not been infected with the virus.^6^ Furthermore, patients suffering from a mental illness reported worsening of their mental health during the pandemic, independent of infection with SARS-CoV-2.^7^ A recent meta-analysis concluded that the prevalence of anxiety and depression in the background population (with unknown COVID-19 status) during the pandemic was >30%,^8^ suggesting that the increased incidence of these diseases is caused by indirect effects of the pandemic. This finding highlights the difficulties with defining the origin of psychiatric sequelae following the COVID-19 pandemic.

To disentangle which neuropsychiatric symptoms of PASC are caused by the infection per se*,* we used a rodent model of the disease. More specifically, we assessed several behavioral readouts associated with psychiatric disorders in K18-hACE2 mice infected with SARS-CoV-2, after recovery from the primary infection. The K18-hACE2 mouse expresses the human angiotensin-converting enzyme 2 (hACE2) receptor, under the control of the cytokeratin-18 (K18) promoter, allowing SARS-CoV-2 to infect cells in both the respiratory tract and the CNS.^9^^,^^10^ This model has proven to be a useful tool in COVID-19 research, for example, when studying neuroinflammation.^11^

SARS-CoV-2 can affect the brain indirectly, through the host’s immune response to the infection, as neuroinflammation is present in many COVID-19 sufferers.^12-14^ In line with that, microgliosis and increased cytokine levels are found in the brains of COVID-19 victims.^15^ Moreover, cytokines are known to activate the kynurenine pathway (KP), which can contribute to the dysregulation of the glutamatergic and monoaminergic systems in psychiatric disorders (see eg,^16-19^). Post-acute cognitive sequelae of COVID-19 have been associated with elevated levels of KP metabolites, and KP metabolites have also been shown to be predictors of depression, anxiety, and stress after COVID-19.^20^^,^^21^

Another possible mechanism contributing to the late psychiatric sequelae of COVID-19 is the dysregulation of the so-called gut-brain axis^22^ as the microbiome is altered in patients suffering from COVID-19.^23^

In summary, evidence shows that COVID-19 can affect the brain, and may lead to long-term psychiatric and/or cognitive deficits. Herein, we used an animal model of COVID-19 to examine whether the neuropsychiatric and cognitive consequences associated with SARS-CoV-2 infection could result from biological consequences of the viral infection.

## MATERIALS AND METHODS

### Animals

Heterozygous male and female K18-hACE C57BL/6 J mice (strain: 2B6.Cg-Tg(K18-ACE2)2Prlmn/J) were obtained from Jackson Laboratory (Stock number: 034860) and bred in-house at Department of Biomedicine (Aarhus University). The animals were group-housed (4-6 per cage) until 1 week before the behavioral experimentation, where they were individually housed due to the sucrose preference test (SPT). They had free access to a standard chow diet and water, with a 12-hour light–dark cycle (lights on 6 AM). The animals were housed in a pathogen-free facility and were weighed daily throughout the investigation. All experiments, except behavior in the phenotyper cages and SPT, were performed solely in the light phase. All procedures were performed in accordance with national and local laws and were approved by the Danish Animal Experiments Inspectorate (Permit number: 2020-15-0201-00726).

### Infection

The animals were 12-14 weeks old at the time of infection. They were anaesthetized with isoflurane and administered 1 × 10^3^ plaque-forming units (p.f.u.) SARS-CoV-2 Alpha B.1.1.7 strain (*n* = 44) or saline (sham, *n* = 29) via intranasal administration. The dose was selected based on prior studies to elicit a robust acute immune response while decreasing mortality risk. The mice were randomly assigned to treatment group. Animals that lost >20% of their initial bodyweight (BW) were euthanized (*n* = 15). Infection was subsequently confirmed using a commercially available ELISA kit measuring SARS-CoV-2 spike protein total antibodies (see section 2.5). Weight loss is a sensitive and quantifiable indicator of general health deterioration and can therefore be used to track disease progression. The animals were categorized as “symptomatic” if they lost more than 2% of their BW in the first 2 weeks following the infection. Animals that lost less than 2% of their BW during the first 2 weeks after the infection were classified as “asymptomatic”. The cut-off value for weight loss was defined a priori as any weight loss exceeding that observed in sham-treated animals following infection, thereby accounting for the effects of the infection procedure itself. As sham-treated animals did not exceed a weight loss of 2%, this threshold was applied. This approach ensured that only weight loss exceeding normal procedural effects was considered.

### Behavioral Testing

**Figure 1:**
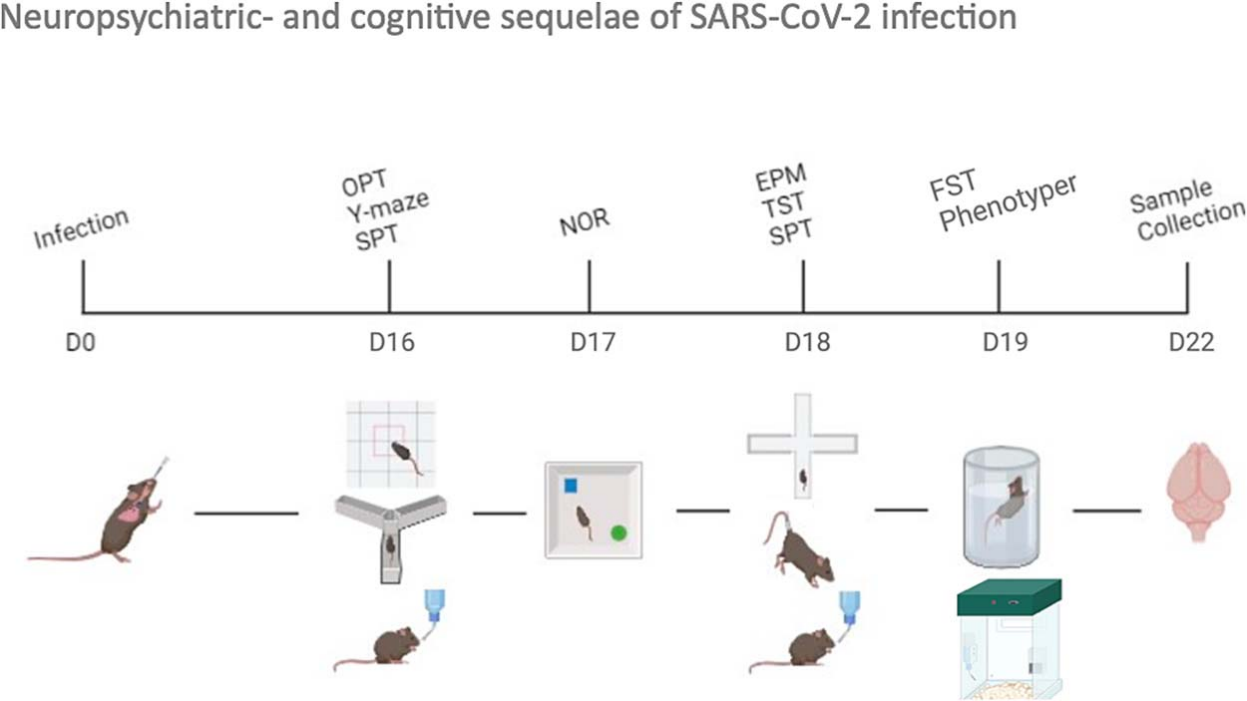
Timeline of behavioral experiments. Mice received intranasal administration of virus or vehicle on day 0. After recovering from infection, they were subsequently tested from day 16 to 19 (D16-D19) in the following behavioral paradigms: Open-field test (OFT), Y-maze, sucrose preference test (SPT), novel object recognition (NOR), elevated plus-maze (EPM), tail suspension test (TST), and forced swim test (FST), followed by the phenotyper. Tissue was harvested on day 22.

Behavioral testing started 16 days after SARS-CoV-2 infection. At this time point, the mice had recovered from the acute infection, and BW was stable (f sham *n* = 16, m sham *n* = 13, f infected *n* = 18 (*n* = 12 symptomatic, *n* = 6 asymptomatic), m infected *n* = 11 (*n* = 8 symptomatic, *n* = 3 asymptomatic)). See Figure 1 for an overview of the behavioral experiments. The order of the behavioral tests was selected to progress from least stressful and requiring highest cognitive functioning to more stressful but requiring less cognitive function. Animals underwent the behavioral tests in a treatment randomized order, and the experimenter was blind for treatment. During the subsequent manual scoring, the observer was blind to treatment and sex.

#### Open Field Test

Spontaneous locomotor activity was examined using the Open Field Test (OFT). Each animal was placed in the center of the open field arenas (49 × 49 cm grey Plexiglas boxes) in a dimly lit room (40-50 lux). Total distance moved over 20 minutes was recorded using EthoVision XT 17.5 tracking software (Noldus, The Netherlands).

#### Forced Swim Test

The Forced Swim Test (FST) was performed to examine despair-like behavior. The FST was performed in a dimly lit room (40-50 lux). Each mouse was placed in a transparent cylinder (25 cm high, ø 10 cm) filled with water (temp. 25$\pm$0,5 °C).^24^ The test lasted 6 minutes, and the total duration of immobility was subsequently scored manually.^25^

#### Tail Suspension Test

The Tail Suspension Test (TST) is another tool to screen for despair-like behaviors in mice.^26^ The test was performed in a box (21 × 20 × 40 cm) with 1 open side (40-50 lux). The box had a 10 cm metal pole protruding the back wall. The mouse was suspended from the tail by placing tape on the tail, which was fixed over the metal pole. The test lasted 10 minutes. The duration of immobility throughout the test was subsequently scored manually.

#### Sucrose Preference Test

The SPT was used as a measure of anhedonia-like behavior. The test was performed in the animal’s home cage. The mice were presented with a choice of 2 bottles: 1 containing tap water and the other containing a 2% sucrose solution.^27^ The test lasted 12 hours and was performed during the dark cycle. The sucrose preference was evaluated every second day for 4 days. Sucrose preference was calculated as follows:


\begin{align*}& Sucrose\ preference\ \left(\%\right)\ =\frac{sucrose\ consumption\ (g)}{sucrose\ + water\ consumption\ (g)}*100\% \end{align*}

The average sucrose preference for the 4 days was used in subsequent analyses.

#### Y-Maze

Spontaneous alternation in the Y-maze was used as a measure of working memory performance.^28^ The maze consisted of 3 identical arms in dark Plexiglas (each arm: h:15 cm, l:40 cm, w:8 cm; 40-50 lux). The animal was placed at the end of 1 arm of the maze and allowed to freely explore all arms for 5 minutes. The sequence of arm entries was recorded, and the percent correct alternations (when the animal enters 3 different arms consecutively) was subsequently calculated.


\begin{align*} Correct\ alternations\ \left(\%\right)=\frac{number\ of\ correct\ alternations}{total\ number\ of\ alternations}*100\% \end{align*}

#### Novel Object Recognition

Novel Object Recognition (NOR) was performed to assess recognition memory in the mice.^29^ The test was conducted in the open field arena (see above) and consisted of an encoding session and a test session conducted 1 hour after encoding. The animal was placed in the arena for 5 minutes for each session with 2 objects. During the encoding session, the objects were identical, whereas, for the test session, 1 of the objects was changed to a novel object. Time spent exploring the objects was scored manually. Preference for the novel object was calculated as follows:


\begin{align*}& Novel\ object\ recognition\ \left(\%\right)\\&\quad =\frac{time\ exploring\ novel\ object}{time\ exploring\ novel+ familiar\ object}*100\% \end{align*}

#### Elevated Plus Maze

The Elevated Plus Maze (EPM) was used as a measure of anxiety-like behavior.^30^ The maze was a platform shaped as a plus with 2 open arms (29 × 6 cm) and 2 closed arms (29 × 5 cm, with 15 cm high walls). The maze was elevated 44 cm from the ground, and the light intensity was 100 lux in the open arms. The mouse was allowed to explore freely for 5 minutes. Time spent in open versus closed arms was analyzed using EthoVision. Percent time spent in the open arms was calculated as:


\begin{align*}& Time\ in\ open\ arms\ \left(\%\right)\ =\frac{time\ in\ open\ arms}{time\ in\ open\ + closed\ arms}*100\% \end{align*}

#### Phenotyper

Spontaneous home cage behavior was recorded using Noldus Phenotypers. Following the last behavioral test, the animals were placed in phenotypers for 18 hours, and their behavior was tracked throughout this period. Spontaneous behavior, including movement, time spent in the shed, and time spent eating and drinking, was recorded in EthoVision.

### Sample Collection

Three days after the final behavioral test (ie, 22 days post-inoculation), the animals were sacrificed by decapitation, and samples were collected. Brain regions of interest and caecum content were isolated on ice, snap-frozen on dry ice, and stored at -80 °C. Blood was allowed to coagulate at room temperature and thereafter centrifuged at 4 °C for 10 minutes at 1300 g. Serum was subsequently collected and stored at -80 °C.

### SARS-CoV-2 Spike Protein Total Antibody ELISA

SARS-CoV-2 spike protein total antibodies were determined using a commercially available ELISA kit (E-EL-E607, Elabscience, Texas, United States). All samples and controls were run in duplicates, and a threshold optical density reading was used to determine seropositivity.

### Cytokine Detection

Cytokines, chemokines, and adhesion molecules in the hypothalamus were measured using a proteome profiler cytokine array kit Panel A (Bio-techne, Dublin, Ireland). Briefly, tissue was homogenized in 10 × PBS + Complete protease inhibitor cocktail (Roche, Germany) on a Precellys Evolution (Bertin Corp, Maryland, United States). Following homogenization, 0.8% Triton-X 100 was added. The supernatant was collected by centrifugation (10.000 g, 4 °C, 5 minutes), and total protein content was determined using Pierce BCA Protein Assay Kit (Thermo Scientific, IL, United States). 200 μg protein was analyzed following manufacturer’s instructions. The expression of cytokines and chemokines was determined using IRDye 800CW Streptavidin (Li-Cor Biosciences, Bad Homburg, Germany) and visualized on an Odyssey Imager with resolution 84 μm and intensity 5.

### Kynurenine Pathway Metabolites

Tryptophan (Trp), kynurenine (KYN), picolinic acid (PIC), quinolinic acid (QUIN), kynurenic acid (KYNA), and 3-hydroxy-kynurenine (3-HK) in the mouse frontal cortex were quantified using ultra-performance liquid chromatography coupled with tandem mass spectrometry (UPLC-MS/MS) on a Xevo-XS triple-quadrupole mass spectrometer equipped with a Z-spray electrospray interface and a Waters Acquity UPLC I-Class FTN system. See Schwieler, Trepci, Krzyzanowski, Hermansson, Granqvist, Piehl, Venckunas, Brazaitis, Kamandulis, Lindqvist, Jones, Erhardt and Brundin^31^ for full details on the method.

### Monoamine Neurotransmitters

Monoamine neurotransmitters and their metabolites were examined in the mouse hippocampus using ultra-high performance liquid chromatography (uHPLC), as described in Liebenberg, Jensen, Larsen, Kousholt, Pereira, Fischer and Wegener.^32^ Briefly, samples were homogenized I HClO_4_ using Precellys tissue homogenizer (Bertin Technologies, France) and filtered through Costar cellulose acetate filter tubes (0.22 μm; Corning Inc., Corning, NY, United States). Noradrenaline (NA), dopamine (DA), 3,4-dihydroxyphenylacetic acid (DOPAC), homovanillic acid (HVA), 5-hydroxytryptamine (5-HT), and 5-hydroxyindoleacetic acid (5-HIAA) were separated on a 150 × 4.6 mm Kinetex 2,6 μm EVO C18 100 Å, Size LC Column (Phenomenex, United States) kept at 28 °C. Detection was carried out using a Thermo Scientific Dionex model 6011RS ultra 2-channel Coulometric Analytical cell (E1: -150 mV: E2: +250 nV vs Pd reference) attached to a Thermo Scientific Dionex Ultimate 3000 uHPLC system while eluting the analytes with a MDTM mobile phase (Thermo Scientific Dionex Test Phase, 70-3829) at a flow rate of 1.5 mL/minute.

### Gut Microbiome

DNA extraction, library preparation, and Shotgun Metagenomic Sequencing were performed on caecum content. DNA extraction was performed by DNAsense, DK sequencing by BMKgene, DE, and Bioinformatics by Biomcare, DK. DNA was extracted from the caecum content using the FastDNA SPIN Kit for Soil, followed by DNA quality evaluation using a combination of Nanodrop, Qubit, and Gel electrophoresis methods. DNA extraction of samples was done using a slightly modified version of the standard protocol for FastDNA Spin kit for Soil (MP Biomedicals, United States) with the following exceptions: 500 μL of sample, 480 μL Sodium Phosphate Buffer, and 120 μL MT Buffer were added to a Lysing Matrix E tube. Bead beating was performed at 6 m/s for 4 × 40 s. DNA concentration was measured using Qubit dsDNA HS/BR Assay kit (Thermo Fisher Scientific, United States).

The genomic DNA was fragmented using an enzyme-based fragmentation with FEA Enzyme Mix, and for library construction, the VAHTS universal Plus DNA Library Prep Kit for Illumina V2 was used. For the constructed library, the Illumina NovaSeq X (Illumina, Santiago, CA, United States) was used for sequencing.

#### Taxonomic Profiling Using MetaPhlAn

MetaPhlAn (Metagenomic Phylogenetic Analysis, Harvard, United States) was used to generate taxonomic profiles. For the identified clades of organisms, we calculated both the relative abundance to sum to 100% for each taxonomic level (excluding the unknown fraction) and their estimated read count. The counts were calculated per clade by estimating the number of reads that should originate from that clade. The calculation considered the coverage of the clades’ markers and the length of the clade’s genome as given by the reference genomes.

### Statistical Analysis

Statistical analysis was performed using IBM SPSS Statistics for macOS 28.0 software (IBM Corp., United States). Charts were made using GraphPad Prism 10.0 for macOS (GraphPad Software, United States), and figures were created using BioRender (BioRender, Canada). Data were stratified after treatment and infection response (sham, symptomatic, and asymptomatic). For comparison of sex and infection response, 2 × 3 factorial ANOVAs were performed with sex (m or f) and infection response (sham, symptomatic, or asymptomatic) as independent variables. Non-normally distributed data were ln-transformed. Post hoc LSD tests were applied when a significant effect of infection response was found. When a significant interaction between sex and infection response was found, post hoc LSD tests were performed. z-scores for cognition and depression were calculated. For cognition, z-scores were calculated for % correct choices in the Y-maze and % time exploring the novel object in NOR. The average of these scores was used as the overall z-score of cognition. For depression, z-scores were calculated for immobility in the FST and TST, as well as for % sucrose intake in the SPT. The average of these 3 scores was used as the z-score for depression. Correlations between behavior and KP metabolites were performed using Pearson Correlation. To examine for False Discovery Rate (FDR), Benjamini-Hochberg procedure was applied (FDR level = 5%) for Cytokine measurements (40 analytes) and KP metabolites (6 analytes).

To assess the association between the gut microbiome and the infection response, we evaluated the overall microbial composition (beta-diversity) using Permutational Multivariate Analysis of Variance (ADONIS) models. Alpha diversity was reported as observed species (richness) and as Shannon diversity, which reflects both richness and evenness of a microbiome community. In each model, we adjusted for sex and performed 1 analysis of each alpha diversity measure. For beta-diversity, we used Bray-Curtis and data at the species level. To estimate significance, the analyses were performed using 999 permutations.

For the analysis of single taxa, we used the DESeq suite in the DESeq2 R package that “Estimate variance–mean dependence in count data from high-throughput sequencing assays and test for differential expression based on a model using the negative binomial distribution” (see details in Love, Huber, and Anders^33^). DESeq2 automatically applies Benjamini-Hochberg FDR correction. SGB abundance was analyzed using the DESeq2 setup to compare infection response groups. To reduce noise, the dataset was first filtered to remove taxa not seen with a count of more than 100 in at least 50% of the samples.

## RESULTS

### SARS-CoV-2 Antibodies

The SARS-CoV-2 spike protein total antibody ELISA showed that 3 animals, which had been infected, did not present any blood antibodies (Supplementary Figure S1). Since previous studies have shown that even mild SARS-CoV-2 infection gives a strong SARS-CoV-2 antibody response in K18-hACE mice,^34^ these animals were excluded from further analysis.

### Body Weight

**Figure 2:**
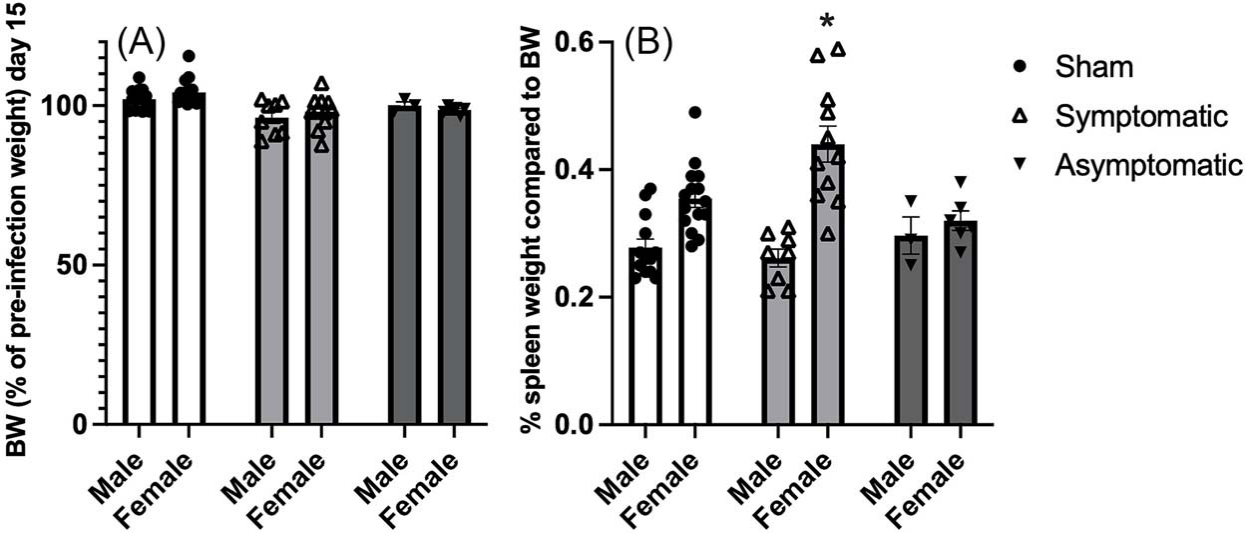
(A) Bodyweight (BW) as % of pre-infection weight reported on recovery, 15 days after infection. (B) Spleen weight relative to BW. Data represent mean +/- SEM. * = *P* < .05 compared to sham-treated females.

On day 15, all animals had recovered from the infection, and no differences were found in BWs between infected and uninfected mice (All F’s < 0.2, see Figure 2*A*).

### Behavior

#### Spontaneous Activity

Spontaneous activity was not affected by SARS-CoV-2 infection, neither measured as total distance moved in the OFT nor activity in the Phenotyper [all F’s < 1.7] (Supplementary Figure S2*A*).

#### Depression-like Behavior

Despair-like behavior was assessed using the FST and the TST. No significant differences were found between infection response groups [all F’s < 0.5]. The SPT was used to measure anhedonia-like behavior. No differences were found here either [all F’s < 1.4, Supplementary Figure S2]. An overall z-score for depression, including FST, TST, and SPT, was performed, and no differences between groups were found [all F’s < 0.8; observed power 0.69] (Supplementary Figure S2).

#### Anxiety-like Behavior

Anxiety-like behavior was measured as time in open arms of the EPM. Previous SARS-CoV-2 infection did not affect the relative time spent in the open arms compared to the closed arms [all F’s < 1.4] (Supplementary Figure S2*F*).

#### Cognition

**Figure 3:**
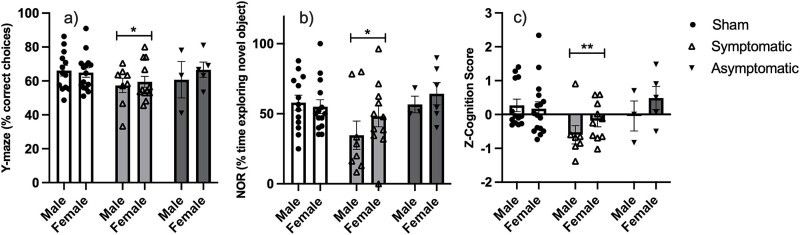
Measures of cognition. (A) % correct choices in the Y-maze, (B) % time exploring the novel object in NOR, C) Z-cognition score. Data represent mean +/- SEM. * = *P* < .05 compared to sham.

Spatial working memory was assessed using the Y-maze, and object recognition memory was assessed using the NOR test. In the Y-maze, an effect of infection response was found [F(2,48) = 3.063, *P* = .050]. Post hoc LSD showed that symptomatic animals differed significantly from sham animals (*P* = .019). Asymptomatic animals did not differ from other groups. Symptomatic animals had a lower percentage of correct choices in the Y-maze (Figure 3*A*), which indicates impaired working memory.

A significant effect of treatment was found for the NOR [F(2, 47) = 3.773, *P* = .030]. Symptomatic animals displayed impaired cognition (Post hoc LSD sham vs Symptoms: *P* = .035; Figure 3*B*).

As an overall measure of cognitive performance, Z-scores for cognition were evaluated. A significant effect of infection response was found [F(2, 49) = 5.474, *P* = .007; observed power 0.56]. Post hoc LSD tests showed that sham-treated mice differed significantly from symptomatic animals (*P* = .005). Symptomatic animals displayed a decreased cognition score (Figure 3*C*).

### Cytokines, Chemokines, and Adhesion Molecules

Of the cytokines, chemokines, and adhesion molecules tested, 6 showed differences between groups. These were interleukin (IL)-1β, CXCL13, intracellular adhesion molecule 1 (ICAM-1), chemokine ligand (CCL)5, tissue inhibitors of metalloproteinases (TIMP)1, and Triggering receptor expressed on myeloid cells (TREM)1. See Supplementary Table S1 for results of all measured molecules.

**Figure 4:**
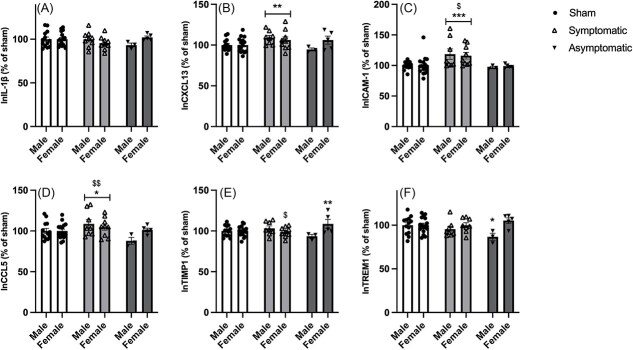
Expression of cytokines and chemokines in the hypothalamus compared to sham-treated animals. (A) IL-1β, (B) CXCL13, (C) ICAM-1, D) CCL5, E) TIMP1, F) TREM1. Data represent mean +/- SEM. * = *P* < .05 compared to sham treated equivalent, ** = *P* < .01 compared to sham, *** = *P* < .001 compared to sham, $ = *P* < .05 compared to asymptomatic infected animals, $$ = *P* < .01 compared to asymtomatic infected animals.

For IL-1β, a tendency toward an interaction between infection response and sex was found [F(2, 48) = 3.018, *P* = .058] (Figure 4*A*).

For CXCL13, a significant effect of infection response was found [F(2, 48) = 4.788, *P* = .013]. Post hoc LSD tests showed that symptomatic but not asymptomatic animals differed from the sham-treated group (*P* = .006 and *P* = .763, respectively), with CXCL13 being elevated in symptomatic animals (Figure 4*B*).

For ICAM-1, an effect of infection response was found [F(2,48) = 6.556, *P* = .001]. Post hoc LSD test showed that symptomatic animals differed from both sham and asymptomatic animals (*P* = .001 and 0.026, respectively). Symptomatic animals displayed a marked increase in ICAM-1 compared to sham and asymptomatic animals (Figure 4*C*).

For CCL5, a significant effect of infection response was found [F(2,48) = 4.797, *P* = .013]. Post hoc tests revealed that symptomatic animals differed from the sham and asymptomatic groups (*P* = .04 and *P* = .007, respectively). CCL5 was elevated in symptomatic animals (Figure 4*D*).

For TIMP1, an interaction between infection response and sex was found [F(2,48) = 5.486, *P* = .007]. Post hoc LSD tests revealed that asymptomatic females differed from sham females (*P* = .005) and that symptomatic females differed from asymptomatic females (*P* = .004). For males, no differences were found [all F’s < 1.7]. Asymptomatic females displayed an increase in TIMP1 (Figure 4*E*).

For TREM1, an interaction between infection response and sex was found [F(2,47) = 4.766, *P* = .013]. After splitting data by sex, a significant difference was found between male sham and male asymptomatic animals (*P* = .021). No other groups differed significantly. As shown in Figure 4*F*, male asymptomatic animals had decreased levels of TREM1 compared to sham.

The observed power was 0.46-0.97, and after FDR correction, only ICAM-1 remained significant (critical FDR threshold = 0.001).

### Monoamines

**Figure 5:**
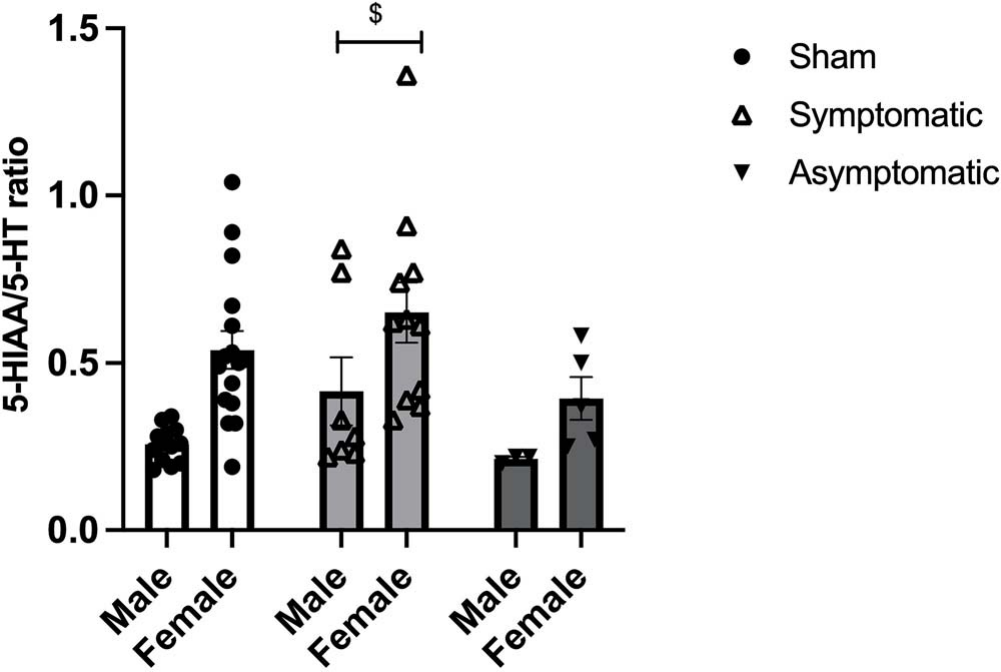
5-HIAA/5-HT ratio in the hippocampus. Data represent mean +/- SEM. $ = *P* < .05 compared to asymptomatic infected animals.

No differences were found for monoamine or monoamine metabolite levels (see Supplementary Table S2). A significant increase in 5-HT metabolism was found (calculated as 5HIAA/5HT) [F(2,48) = 3.197, *P* = .049]. Post hoc LSD test revealed that symptomatic animals differed from asymptomatic (*P* = .021), with symptomatic animals having an increased 5-HT metabolism (Figure 5).

### Kynurenine Pathway Metabolites

Tryptophan and 5 KP metabolites were measured. Four KP metabolites were affected by treatment.

**Figure 6:**
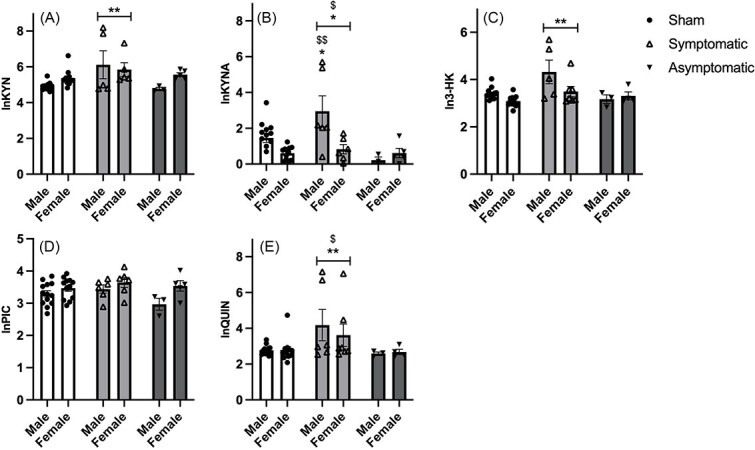
Expression of kynurenine pathway metabolites. (A) Kynurenine, (B) kynurenic acid, (C) 3-hydroxykynurenine, (D) picolinic acid, (E) quinolinic acid. Data represent mean +/- SEM. * = *P* < .05 compared to sham treated equivalent, ** = *P* < .01 compared to sham, $ = *P* < .05 compared to asymptomatic infected animals, $$ = *P* < .01 compared to asymtomatic infected animals.

A significant effect of infection response was found for kynurenine [F(2,37) = 3.780, *P* = .032]. Post hoc LSD tests showed that symptomatic animals differed from sham animals (*P* = .009), with symptomatic animals showing increased expression of kynurenine (Figure 6*A*).

For KYNA, a significant effect of both sex [F(2,37) = 4.120, *P* = .050] and of infection response [F(2,37) = 4.061, *P* = .025], as well as an interaction between sex and infection response [F(2,37) = 4.502, *P* = .018] was found. Post hoc LSD tests showed that symptomatic animals differed from both sham (*P* = .043) and asymptomatic animals (*P* = .020). No differences were found in females, but symptomatic males differed from sham (*P* = .029) and asymptomatic males (*P* = .007) as symptomatic animals showed increased expression of KYNA, which was most pronounced in males (Figure 6*B*).

For 3-HK, a significant effect of infection response was found [F(2,37) = 5.094, *P* = .011]. Post hoc LSD tests revealed that symptomatic animals were significantly different from sham animals (*P* = .006). As can be seen in Figure 6*C* 3-HK levels increased in symptomatic animals.

For PIC, a tendency for an interaction between infection response and sex was found [F(2,37) = 3.158, *P* = .054] (Figure 6*D*).

For QUIN, a significant effect of infection response was found [F(2,37) = 5.835, *P* = .014]. Post hoc LSD tests revealed that symptomatic animals differed from sham (*P* = .007) and asymptomatic animals (*P* = .036). Symptomatic animals displayed increased expression of QUIN compared to both sham and asymptomatic animals (Figure 6*E*).

The observed power for KP metabolites was found to be 0.49-0.79, and after FDR correction, all 4 significant metabolites remained significant (FDR-adjusted threshold = 0.033). Interestingly, KYN, KYNA, 3-HK, and QUIN all correlated negatively with performance in the y-maze (Supplementary Figure S3).

### Gut Microbiome Analysis

Processing the shotgun sequencing data through quality control and microbiome profiling resulted in detection of 592 unique species. When assigning the sequences at higher taxonomic levels, a total of 455 genera, 201 families, 175 orders, 161 classes, and 11 phyla were detected. On average, 33% of reads were unclassified (*n* reads = 18 161 662).

No differences between infection response were found for alpha diversity, neither measured as observed species (richness) nor using the Shannon Index at species level [all t’s < 1.5]. The ADONIS models showed no differences in beta-diversity using Bray-Curtis and data at species level between infection response [all F’s < 0.7] (see Supplementary Figure S4).

**Figure 7:**
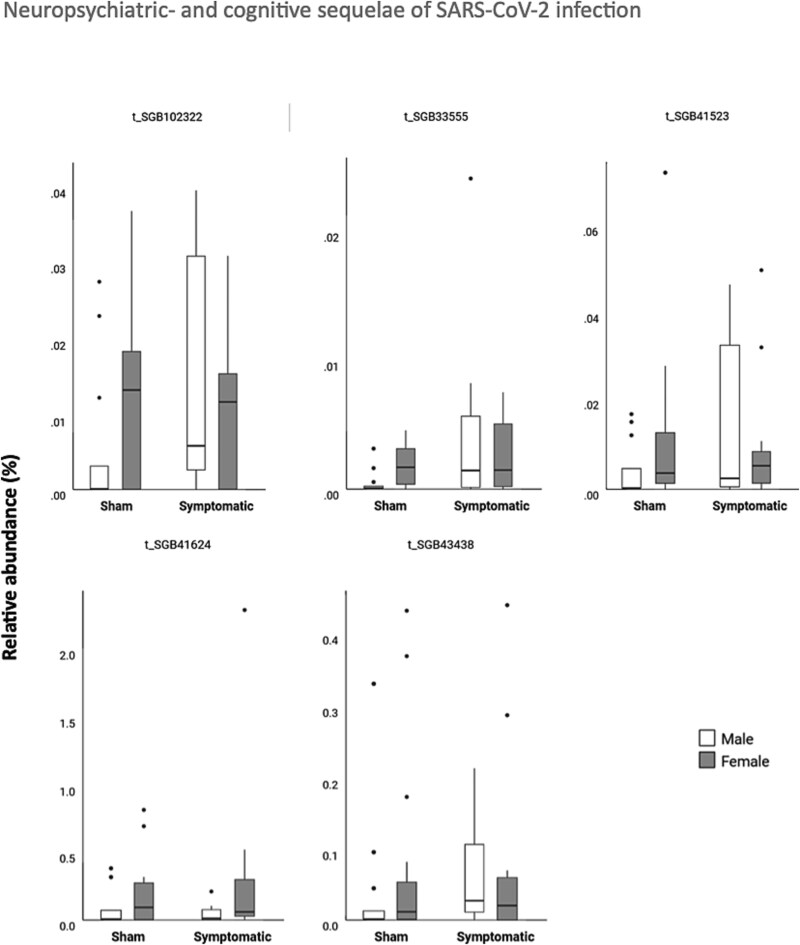
Boxplots showing taxa abundance of the top associated taxa comparing sham to symptomatic animals.

After removal of taxa with counts <100 in >50% of the samples, 208 species-level genome bins (SGBs) were included for analysis. Five single taxa had a *P*-value < .01 and *P*. adj < .2 between sham and symptomatic animals. This *P*.adj was selected to balance the large number of taxa tested against the modest sample sizes. Four taxa were higher in symptomatic animals compared to sham-treated. These were *Parvibacter caeciola* (SGB33555) and 3 firmicutes of undescribed class (SGB102322, SGB41523, and SGB43438). One staphylococcaceae of an undescribed genus was decreased in symptomatic animals compared to controls (SGB41624) (see Figure 7).

**Figure 8:**
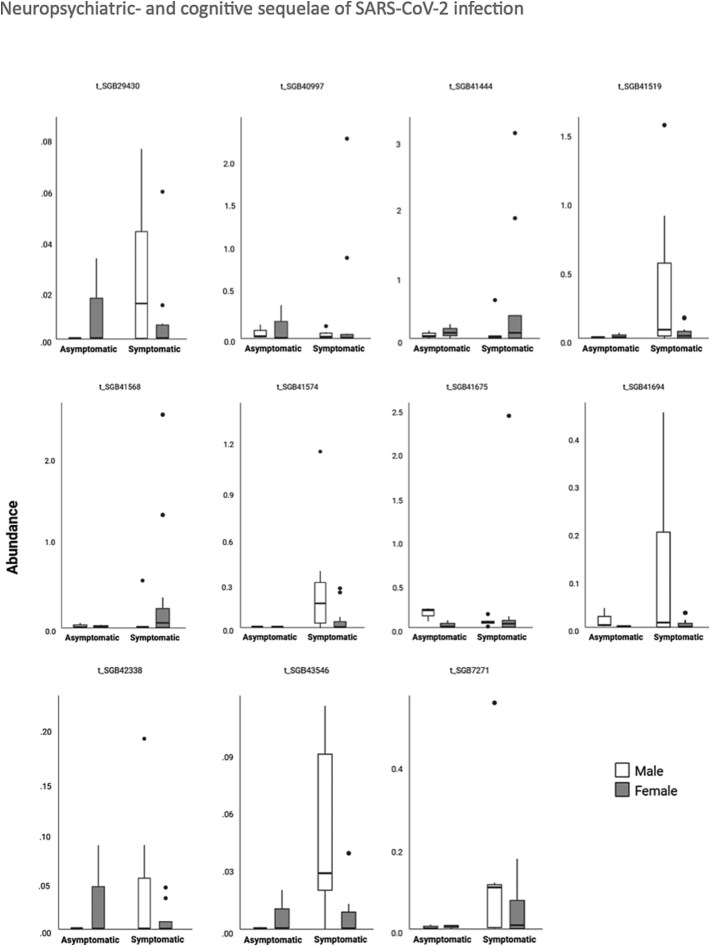
Boxplots showing taxa abundance of the top associated taxa comparing asymptomatic to symptomatic animals.

When comparing asymptomatic to symptomatic animals, 11 taxa differed between the 2 groups [all *P*’s < 0.01 and all *P*.adj < .2]. Nine bacteria were elevated in symptomatic animals, whereas 2 were decreased in symptomatic mice. The elevated bacteria were 4 firmicutes of undescribed class (SGB29430, SGB41519, SGB41694, and SGB42338), a firmicutes of the eubacteriaceae family (SGB41444), a bacteria of unknown phylum (SGB41568), a firmicutes of the oscillospiraceae family (SGB43546), and a firmicutes of the lachnospiraceae family (SGB7271). The 2 bacteria that were decreased in symptomatic animals were a firmicutes of the eubacteriaceae family (SGB40997) and a firmicutes of the clostridiaceae genus (SGB41675) (see Figure 8).

## DISCUSSION

In the current study, we examined behavior related to psychiatric illness and cognitive function to elucidate whether the sequelae reported in patients could be induced by the virus or are more likely to be related to the contextual stress linked to the COVID-19 pandemic. Our results indicate that the behavioral domains related to anxiety- and depression-like behavior were not significantly affected in the infected animals. This could suggest that post-COVID anxiety and depression may be more closely related to the stressors associated with experiencing a pandemic, rather than the direct effects of the viral infection itself. This aligns with the recent study showing that one-third of patients reporting psychiatric sequelae did not have antibodies against SARS-CoV-2.^6^ It should be noted that, while we took measures to minimize carry-over effects when designing the behavioral test battery, such effects can never be entirely ruled out. This limitation is inherent in any study using behavioral test batteries and should be considered when interpreting the results.

In agreement with the human data,^35^ our animal model revealed that memory was impaired in infected animals. The behavioral data, thus, indicate that the cognitive deficits manifested after recovery from acute COVID could be caused by the viral infection per se and that disease severity is highly relevant for the development of these sequelae. It is important to highlight that the K18-hACE2 model is characterized by a higher degree of neuroinvasion than is typically observed in human SARS-CoV-2 infections.^11^ Thus, some of the CNS-related changes reported here may reflect direct consequences of viral replication in the brain, rather than systemic illness alone. This limitation should be considered when extrapolating our findings to the human condition. Despite this, the model remains valuable for identifying potential mechanistic links between viral infection, immune activation, and neurobiological outcomes, while highlighting pathways that warrant further investigation in cohorts with less pronounced neuroinvasion.

A cytokine storm has been suggested to be involved in the sickness behavior and severity in COVID-19 patients.^36^ Post-mortem studies have furthermore revealed an increased expression of cytokines in the brains of COVID-19 victims.^15^ We, therefore, examined if cytokine and chemokine expression would be altered in the hypothalamus to explore whether upregulation of these molecules in a brain region that is central for circadian and emotional regulation, as well as neuroimmune signaling^37-39^ would predict late COVID-19 symptoms.

A tendency for IL-1β to be decreased in asymptomatic animals was found. This may suggest that lower hypothalamic IL-1β levels are linked to reduced sickness and cognitive sequelae, although additional experiments are needed to establish causality and robustness of the result.

Further, symptomatic animals displayed an increase in CXCL13, whereas asymptomatic animals were unaffected. Interestingly, CXCL13 is induced in the brain in different inflammatory conditions^40^ and has been associated with cognitive deficits.^41^ While our findings are consistent with this, the functional implications in the present model remain to be clarified.

Similar to CXCL13, ICAM-1 was upregulated in symptomatic but not asymptomatic animals. ICAM-1 can be upregulated by IL-1^42^^,^^43^ and has previously been shown to predict cognitive impairments in several diseases.^44-46^

CCL5 was upregulated in symptomatic but not asymptomatic animals. CCL5 can activate microglia and impair LTP and neuroplasticity.^47-51^

TIMP1 can be produced by astrocytes^52^^,^^53^ and is positively associated with memory function.^54^ In our current study, asymptomatic females had elevated levels of TIMP1, which may serve as a protective mechanism against cognitive decline.

In asymptomatic male mice, we identified decreased levels of TREM1, a protein receptor expressed on macrophages and microglia and involved in production of several pro-inflammatory cytokines, and increased levels of TREM1 are described in AD.^55^ Asymptomatic males may be protected from cognitive decline by dampened TREM1 levels. Of note, after FDR correction for multiple comparisons, only ICAM-1 remained significant. This limitation should be considered when interpreting the results.

Most dietary tryptophan is metabolized through the KP, making it possible that an overactivation of the KP may lead to serotonin depletion.^56^ We measured serotonin in the hippocampus and found an increase in 5-HT metabolism. The hippocampus was chosen due to its well-described role in memory and affective disorders.^57^^,^^58^ Of course, cross-domain comparisons must be done with caution, as cytokines were measured in the hypothalamus, KP metabolites in the frontal cortex, and monoamines in the hippocampus. However, given that these regions are central nodes in neuroimmune and neurochemical regulation, the findings should still provide insights into brain-wide processes.

In the current study, we examined the KP in the frontal cortex of the mouse. The frontal cortex was chosen because of the role of this brain area in affective behavior and memory.^59-61^ We found alterations in 4 out of 5 measured metabolites of the KP. Thus, KYN, KYNA, 3-HK, and QUIN were upregulated in symptomatic animals, which may be related to the behavioral deficits found in the current study. In schizophrenia patients and AD, an overactivation of the KP has been linked to cognitive deficits.^62-64^

Together, these results indicate that the KP is associated with the cognitive deficits found in the current study, potentially activated by cytokines. The KP could, therefore, be an essential target in COVID-19 sequelae and possibly serve as a biomarker for cognitive sequelae of COVID-19. It should be noted that we cannot exclude the possibility that the behavioral tests activated the HPA-axis differently across groups, which could, theoretically, influence the KP response. However, most studies indicate that corticosterone levels normalize within 24 hours after acute stressors such as the FST (see eg,^65^^,^^66^). Future studies should directly examine corticosterone dynamics in SARS-Co-V2-infected animals compared to controls following behavioral stressors.

Gut microbiome alterations and gut barrier dysfunction have been reported for COVID-19 patients, and it has been suggested that these abnormalities are associated with host immune homeostasis.^67^ We, therefore, examined the microbiome but found no differences in alpha or beta diversity, whereas 5 single taxa differed when comparing sham to symptomatic animals. Four of these were elevated in symptomatic animals compared to sham-treated. One of these was *P. caeciola,* which has been identified as a pathobiont for murine intestinal inflammation.^68^ Parvibacter has also been shown to potentially be associated with the KP.^69^ Three firmicutes of undescribed class were also elevated. These could possibly be pathogenic. One Staphylococcaceae of undescribed genus was found to be decreased in symptomatic animals compared to controls.

When comparing asymptomatic to symptomatic animals, 11 single taxa differed between the 2 groups. Nine bacteria were elevated in symptomatic animals, whereas 2 were decreased. The elevated bacteria were 4 firmicutes of undescribed class, a firmicute of the Eubacteriaceae family, a bacterium of unknown phylum, a firmicute of the Oscillospiraceae family, and a firmicute of the Lachnospiraceae family. The 2 bacteria that were decreased in symptomatic animals were a firmicute of the Eubacteriaceae family and a firmicute of the Clostridiaceae genus. These results suggest that the altered composition of firmicutes bacteria is either protective against acute symptoms of SARS-CoV-2 infection or the composition of firmicutes is altered by SARS-CoV-2 infection, leading to more severe acute symptoms of COVID-19 as well as post-acute symptomatology. Interestingly, firmicutes composition has previously been associated with cognitive functioning in older adults, and this was suggested to be related to an inflammatory state.^70^

In summary, we found that previous SARS-CoV-2 infection resulted in behavioral deficits related to cognition but not anxiety- or depression-like behavior. This could imply that the SARS-CoV-2 infection causes PASC cognitive deficits, whereas PASC anxiety and depression may be associated with contextual stress factors rather than the viral infection itself. Our results furthermore suggest that the cognitive deficits were affected by the severity of the acute disease. We also showed that some cytokines and 4 out of 5 measured metabolites of the KP were upregulated in symptomatic animals, indicating that these molecules may be substrates underlying late COVID cognitive symptoms. This suggests a potential for these molecules as biomarkers for the development of cognitive sequelae and possibly also as targets for pharmacological interventions. Interestingly, we found interactions with sex for TIMP1, TREM1, and KYNA. This should be examined further in higher-powered studies, as it may be related to the sex specific findings in PASC.^5^ Furthermore, we showed that the microbiome, particularly the composition of Firmicutes, was altered in animals suffering from late COVID symptoms. The microbiota may, therefore, also be a target for future treatment strategies for late COVID.

A limitation of this study is that the observed power for detecting effects was 0.49-0.79 (α = 0.05), increasing the risk of Type II errors. This should be considered when interpreting these exploratory results, and future, higher-powered studies should explore the present results further. Additionally, the current experiments were performed 3 weeks following infection. Future studies will determine the duration and persistence of the observed behavioral deficits.

## Supplementary Material

SupplFigureS1_310725_pyaf072

SupplFigureS2_310725_pyaf072

SupplFigureS3_310725_pyaf072

SupplFigureS4_310725_pyaf072

SupplTableS1_310725_pyaf072

SupplTableS2_310725_pyaf072

## Data Availability

Data are available to qualified investigators upon request.

## References

[1] Gavriatopoulou M, Korompoki E, Fotiou D, et al. Organ-specific manifestations of COVID-19 infection. *Clin Exp Med*. 2020;20:493-506. 10.1007/s10238-020-00648-x 32720223 PMC7383117

[2] Helms J, Kremer S, Merdji H, et al. Neurologic features in severe SARS-CoV-2 infection. *N Engl J Med*. 2020;382:2268-2270. 10.1056/NEJMc2008597 32294339 PMC7179967

[3] Egbert AR, Cankurtaran S, Karpiak S. Brain abnormalities in COVID-19 acute/subacute phase: a rapid systematic review. *Brain Behav Immun*. 2020;89:543-554. 10.1016/j.bbi.2020.07.014 32682993 PMC7366124

[4] Hu Y, Chen Y, Zheng Y, et al. Factors related to mental health of inpatients with COVID-19 in Wuhan. *China Brain Behav Immun*. 2020;89:587-593. 10.1016/j.bbi.2020.07.016 32681866 PMC7362867

[5] Schou TM, Joca S, Wegener G, Bay-Richter C. Psychiatric and neuropsychiatric sequelae of COVID-19 - a systematic review. *Brain Behav Immun*. 2021;97:328-348. 10.1016/j.bbi.2021.07.018 34339806 PMC8363196

[6] Fogh K, Larsen TG, Hansen CB, et al. Self-reported long COVID and its association with the presence of SARS-CoV-2 antibodies in a Danish cohort up to 12 months after infection. *Microbiol Spectr*. 2022;10:e0253722. 10.1128/spectrum.02537-22 36350150 PMC9769646

[7] Kolbaek P, Jefsen OH, Speed M, Ostergaard SD. Mental health of patients with mental illness during the COVID-19 pandemic lockdown: a questionnaire-based survey weighted for attrition. *Nord J Psychiatry*. 2022;76:338-347. 10.1080/08039488.2021.1970222 34533424

[8] Salari N, Hosseinian-Far A, Jalali R, et al. Prevalence of stress, anxiety, depression among the general population during the COVID-19 pandemic: a systematic review and meta-analysis. *Glob Health*. 2020;16:57. 10.1186/s12992-020-00589-w PMC7338126 32631403

[9] McCray PB Jr, Pewe L, Wohlford-Lenane C, et al. Lethal infection of K18-hACE2 mice infected with severe acute respiratory syndrome coronavirus. *J Virol*. 2007;81:813-821. 10.1128/JVI.02012-06 17079315 PMC1797474

[10] Winkler ES, Bailey AL, Kafai NM, et al. SARS-CoV-2 infection of human ACE2-transgenic mice causes severe lung inflammation and impaired function. *Nat Immunol*. 2020;21:1327-1335. 10.1038/s41590-020-0778-2 32839612 PMC7578095

[11] Kumari P, Rothan HA, Natekar JP, et al. Neuroinvasion and encephalitis following intranasal inoculation of SARS-CoV-2 in K18-hACE2 mice. *Viruses.* 2021;13:132. 10.3390/v13010132 PMC7832889 33477869

[12] Divani AA, Andalib S, Di Napoli M, et al. Coronavirus disease 2019 and stroke: clinical manifestations and pathophysiological insights. *J Stroke Cerebrovasc Dis*. 2020;29:104941. 10.1016/j.jstrokecerebrovasdis.2020.104941 32689643 PMC7214348

[13] Muccioli L, Pensato U, Cani I, Guarino M, Cortelli P, Bisulli F. COVID-19-associated encephalopathy and cytokine-mediated Neuroinflammation. *Ann Neurol*. 2020;88:860-861. 10.1002/ana.25855 32715524

[14] Pilotto A, Odolini S, Masciocchi S, et al. Steroid-responsive encephalitis in coronavirus disease 2019. *Ann Neurol*. 2020;88:423-427. 10.1002/ana.25783 32418288 PMC7276848

[15] Soung AL, Vanderheiden A, Nordvig AS, et al. COVID-19 induces CNS cytokine expression and loss of hippocampal neurogenesis. *Brain.* 2022;145:4193-4201. 10.1093/brain/awac270 36004663 PMC9452175

[16] Bay-Richter C, Linderholm KR, Lim CK, et al. A role for inflammatory metabolites as modulators of the glutamate N-methyl-D-aspartate receptor in depression and suicidality. *Brain Behav Immun*. 2015;43:110-117. 10.1016/j.bbi.2014.07.012 25124710

[17] Savitz J . The kynurenine pathway: a finger in every pie. *Mol Psychiatry*. 2020;25:131-147. 10.1038/s41380-019-0414-4 30980044 PMC6790159

[18] Pocivavsek A, Schwarcz R, Erhardt S. Neuroactive kynurenines as pharmacological targets: new experimental tools and exciting therapeutic opportunities. *Pharmacol Rev*. 2024;76:978-1008. 10.1124/pharmrev.124.000239 39304346 PMC11549936

[19] Sellgren CM, Kegel ME, Bergen SE, et al. A genome-wide association study of kynurenic acid in cerebrospinal fluid: implications for psychosis and cognitive impairment in bipolar disorder. *Mol Psychiatry*. 2016;21:1342-1350. 10.1038/mp.2015.186 26666201 PMC4965332

[20] Cysique LA, Jakabek D, Bracken SG, et al. The kynurenine pathway relates to post-acute COVID-19 objective cognitive impairment and PASC. *Ann Clin Transl Neurol*. 2023;10:1338-1352. 10.1002/acn3.51825 37318955 PMC10424655

[21] Kucukkarapinar M, Yay-Pence A, Yildiz Y, et al. Psychological outcomes of COVID-19 survivors at sixth months after diagnose: the role of kynurenine pathway metabolites in depression, anxiety, and stress. *J Neural Transm (Vienna)*. 2022;129:1077-1089. 10.1007/s00702-022-02525-1 35796878 PMC9261222

[22] Mayer EA, Tillisch K, Gupta A. Gut/brain axis and the microbiota. *J Clin Invest*. 2015;125:926-938. 10.1172/JCI76304 25689247 PMC4362231

[23] Yamamoto S, Saito M, Tamura A, Prawisuda D, Mizutani T, Yotsuyanagi H. The human microbiome and COVID-19: a systematic review. *PLoS One*. 2021;16:e0253293. 10.1371/journal.pone.0253293 34161373 PMC8221462

[24] Petit-Demouliere B, Chenu F, Bourin M. Forced swimming test in mice: a review of antidepressant activity. *Psychopharmacology*. 2005;177:245-255. 10.1007/s00213-004-2048-7 15609067

[25] Castagne V, Moser P, Roux S, Porsolt RD. Rodent models of depression: forced swim and tail suspension behavioral despair tests in rats and mice. *Curr Protoc Pharmacol*. 2010;**49**:5.8.1-5.8.4. 10.1002/0471141755.ph0508s49 22294373

[26] Cryan JF, Mombereau C, Vassout A. The tail suspension test as a model for assessing antidepressant activity: review of pharmacological and genetic studies in mice. *Neurosci Biobehav Rev*. 2005;29:571-625. 10.1016/j.neubiorev.2005.03.009 15890404

[27] Eagle A, Mazei-Robison M, Robison A. Sucrose preference test to measure stress-induced. *Bio-protocol.* 2016;6:e1822.

[28] Sarter M, Bodewitz G, Stephens DN. Attenuation of scopolamine-induced impairment of spontaneous alteration behaviour by antagonist but not inverse agonist and agonist beta-carbolines. *Psychopharmacology*. 1988;94:491-495. 10.1007/BF00212843 2836875

[29] Antunes M, Biala G. The novel object recognition memory: neurobiology, test procedure, and its modifications. *Cogn Process*. 2012;13:93-110. 10.1007/s10339-011-0430-z 22160349 PMC3332351

[30] Rodgers RJ, Dalvi A. Anxiety, defence and the elevated plus-maze. *Neurosci Biobehav Rev*. 1997;21:801-810. 10.1016/s0149-7634(96)00058-9 9415905

[31] Schwieler L, Trepci A, Krzyzanowski S, et al. A novel, robust method for quantification of multiple kynurenine pathway metabolites in the cerebrospinal fluid. *Bioanalysis.* 2020;12:379-392. 10.4155/bio-2019-0303 32209024 PMC9472175

[32] Liebenberg N, Jensen E, Larsen ER, et al. A preclinical study of casein Glycomacropeptide as a dietary intervention for acute mania. *Int J Neuropsychopharmacol*. 2018;21:473-484. 10.1093/ijnp/pyy012 29726996 PMC5932479

[33] Love MI, Huber W, Anders S. Moderated estimation of fold change and dispersion for RNA-seq data with DESeq2. *Genome Biol*. 2014;15:550. 10.1186/s13059-014-0550-8 25516281 PMC4302049

[34] Sriramula S, Theobald D, Parekh RU, Akula SM, O'Rourke DP, Eells JB. Emerging role of Kinin B1 receptor in persistent neuroinflammation and neuropsychiatric symptoms in mice following recovery from SARS-CoV-2 infection. *Cells.* 2023;12:2107. 10.3390/cells12162107 PMC10453171 37626917

[35] Ceban F, Ling S, Lui LMW, et al. Fatigue and cognitive impairment in post-COVID-19 syndrome: a systematic review and meta-analysis. *Brain Behav Immun*. 2022;101:93-135. 10.1016/j.bbi.2021.12.020 34973396 PMC8715665

[36] Hu B, Huang S, Yin L. The cytokine storm and COVID-19. *J Med Virol*. 2021;93:250-256. 10.1002/jmv.26232 32592501 PMC7361342

[37] Burdakov D, Peleg-Raibstein D. The hypothalamus as a primary coordinator of memory updating. *Physiol Behav*. 2020;223:112988. 10.1016/j.physbeh.2020.112988 32485184

[38] Bao AM, Meynen G, Swaab DF. The stress system in depression and neurodegeneration: focus on the human hypothalamus. *Brain Res Rev*. 2008;57:531-553. 10.1016/j.brainresrev.2007.04.005 17524488

[39] Fischer S . The hypothalamus in anxiety disorders. *Handb Clin Neurol*. 2021;180:149-160. 10.1016/B978-0-12-820107-7.00009-4 34225926

[40] Huber AK, Irani DN. Targeting CXCL13 during Neuroinflammation. *Adv Neuroimmune Biol*. 2015;6:1-8. 10.3233/NIB-150101 26855687 PMC4743661

[41] Shen Y, Zhang Y, Chen L, et al. Chemokine CXCL13 acts via CXCR5-ERK signaling in hippocampus to induce perioperative neurocognitive disorders in surgically treated mice. *J Neuroinflammation*. 2020;17:335. 10.1186/s12974-020-02013-x 33161894 PMC7648984

[42] Lebedeva T, Dustin ML, Sykulev Y. ICAM-1 co-stimulates target cells to facilitate antigen presentation. *Curr Opin Immunol*. 2005;17:251-258. 10.1016/j.coi.2005.04.008 15886114

[43] Burne MJ, Elghandour A, Haq M, et al. IL-1 and TNF independent pathways mediate ICAM-1/VCAM-1 up-regulation in ischemia reperfusion injury. *J Leukoc Biol*. 2001;70:192-198. 10.1189/jlb.70.2.192 11493610

[44] Pospelova M, Krasnikova V, Fionik O, et al. Adhesion molecules ICAM-1 and PECAM-1 as potential biomarkers of central nervous system damage in women breast cancer survivors. *Pathophysiology.* 2022;29:52-65. 10.3390/pathophysiology29010006 35366289 PMC8952280

[45] Gregory MA, Manuel-Apolinar L, Sanchez-Garcia S, et al. Soluble intercellular adhesion Molecule-1 (sICAM-1) as a biomarker of vascular cognitive impairment in older adults. *Dement Geriatr Cogn Disord*. 2019;47:243-253. 10.1159/000500068 31408858

[46] Janelidze S, Mattsson N, Stomrud E, et al. CSF biomarkers of neuroinflammation and cerebrovascular dysfunction in early Alzheimer disease. *Neurology.* 2018;91:e867-e877. 10.1212/WNL.0000000000006082 30054439 PMC6133624

[47] Skuljec J, Sun H, Pul R, et al. CCL5 induces a pro-inflammatory profile in microglia in vitro. *Cell Immunol*. 2011;270:164-171. 10.1016/j.cellimm.2011.05.001 21620385

[48] Festa BP, Siddiqi FH, Jimenez-Sanchez M, et al. Microglial-to-neuronal CCR5 signaling regulates autophagy in neurodegeneration. *Neuron.* 2023;111:2021-2037 e12. 10.1016/j.neuron.2023.04.006 37105172

[49] Shen Y, Zhou M, Cai D, et al. CCR5 closes the temporal window for memory linking. *Nature.* 2022;606:146-152. 10.1038/s41586-022-04783-1 35614219 PMC9197199

[50] Zhou M, Greenhill S, Huang S, et al. CCR5 is a suppressor for cortical plasticity and hippocampal learning and memory. *Elife.* 2016;20:5. 10.7554/eLife.20985 PMC5213777 27996938

[51] Greco GA, Rock M, Amontree M, et al. CCR5 deficiency normalizes TIMP levels, working memory, and gamma oscillation power in APOE4 targeted replacement mice. *Neurobiol Dis*. 2023;179:106057. 10.1016/j.nbd.2023.106057 36878326 PMC10291850

[52] Schoeps B, Fradrich J, Kruger A. Cut loose TIMP-1: an emerging cytokine in inflammation. *Trends Cell Biol*. 2023;33:413-426. 10.1016/j.tcb.2022.08.005 36163148

[53] Dhar A, Gardner J, Borgmann K, Wu L, Ghorpade A. Novel role of TGF-beta in differential astrocyte-TIMP-1 regulation: implications for HIV-1-dementia and neuroinflammation. *J Neurosci Res*. 2006;83:1271-1280. 10.1002/jnr.20787 16496359 PMC3820372

[54] Chaillan FA, Rivera S, Marchetti E, et al. Involvement of tissue inhibition of metalloproteinases-1 in learning and memory in mice. *Behav Brain Res*. 2006;173:191-198. 10.1016/j.bbr.2006.06.020 16860884 PMC2659720

[55] Liu YS, Yan WJ, Tan CC, et al. Common variant in TREM1 influencing brain amyloid deposition in mild cognitive impairment and Alzheimer's disease. *Neurotox Res*. 2020;37:661-668. 10.1007/s12640-019-00105-y 31721052

[56] Hoglund E, Overli O, Winberg S. Tryptophan metabolic pathways and brain serotonergic activity: a comparative review. *Front Endocrinol (Lausanne)*. 2019;10:158. 10.3389/fendo.2019.00158 31024440 PMC6463810

[57] Sheline YI, Mittler BL, Mintun MA. The hippocampus and depression. *Eur Psychiatry*. 2002;17:300-305. 10.1016/s0924-9338(02)00655-7 15177085

[58] Bannerman DM, Rawlins JN, McHugh SB, et al. Regional dissociations within the hippocampus--memory and anxiety. *Neurosci Biobehav Rev*. 2004;28:273-283. 10.1016/j.neubiorev.2004.03.004 15225971

[59] Moscovitch M, Winocur G. The Fontal Cortex and Working with Memory. In DT Stuss & RT Knight (Eds.), Principles of frontal lobe function. Oxford University Press. 2002;12:188-209.

[60] Kolb B . Functions of the frontal cortex of the rat: a comparative review. *Brain Res*. 1984;320:65-98. 10.1016/0165-0173(84)90018-3 6440660

[61] Kolk SM, Rakic P. Development of prefrontal cortex. *Neuropsychopharmacology.* 2022;47:41-57. 10.1038/s41386-021-01137-9 34645980 PMC8511863

[62] Sapienza J, Spangaro M, Guillemin GJ, Comai S, Bosia M. Importance of the dysregulation of the kynurenine pathway on cognition in schizophrenia: a systematic review of clinical studies. *Eur Arch Psychiatry Clin Neurosci*. 2023;273:1317-1328. 10.1007/s00406-022-01519-0 36460745

[63] Giil LM, Midttun O, Refsum H, et al. Kynurenine pathway metabolites in Alzheimer's disease. *J Alzheimer's Dis*. 2017;60:495-504. 10.3233/JAD-170485 28869479

[64] Liang Y, Xie S, He Y, et al. Kynurenine pathway metabolites as biomarkers in Alzheimer's disease. *Dis Markers*. 2022;2022:9484217. 10.1155/2022/9484217 35096208 PMC8791723

[65] Bouwknecht JA, van der Gugten J, Hijzen TH, Maes RA, Hen R, Olivier B. Corticosterone responses in 5-HT1B receptor knockout mice to stress or 5-HT1A receptor activation are normal. *Psychopharmacology*. 2001;153:484-490. 10.1007/s002130000598 11243496

[66] Garcia A, Marti O, Valles A, Dal-Zotto S, Armario A. Recovery of the hypothalamic-pituitary-adrenal response to stress. Effect of stress intensity, stress duration and previous stress exposure. *Neuroendocrinology.* 2000;72:114-125. 10.1159/000054578 10971146

[67] Sun Z, Song ZG, Liu C, et al. Gut microbiome alterations and gut barrier dysfunction are associated with host immune homeostasis in COVID-19 patients. *BMC Med*. 2022;20:24. 10.1186/s12916-021-02212-0 35045853 PMC8769945

[68] Clavel T, Charrier C, Wenning M, Haller D. Parvibacter caecicola gen. Nov., sp. nov., a bacterium of the family Coriobacteriaceae isolated from the caecum of a mouse. *Int J Syst Evol Microbiol*. 2013;63:2642-2648. 10.1099/ijs.0.045344-0 23291890

[69] Zhang Z, Liu J, Li M, et al. Lactobacillus rhamnosus encapsulated in alginate/chitosan microgels manipulates the gut microbiome to ameliorate salt-induced Hepatorenal injury. *Front Nutr*. 2022;9:872808. 10.3389/fnut.2022.872808 35495927 PMC9047548

[70] Manderino L, Carroll I, Azcarate-Peril MA, et al. Preliminary evidence for an association between the composition of the gut microbiome and cognitive function in neurologically healthy older adults. *J Int Neuropsychol Soc*. 2017;23:700-705. 10.1017/S1355617717000492 28641593 PMC6111127

