# Supplementary material for: Neuropsychiatric- and cognitive post-acute sequelae of SARS-CoV-2 infection – evidence from K18-hACE C57BL/6 J mice
Source: Int J Neuropsychopharmacol. 2025 Sep 30;28(10):pyaf072. doi: 10.1093/ijnp/pyaf072 (PMC12542986; doi:10.1093/ijnp/pyaf072)
Supplement: SupplFigureS1_310725_pyaf072 [file supplfigures1_310725_pyaf072.pdf]

SARS-CoV-2 antibodies

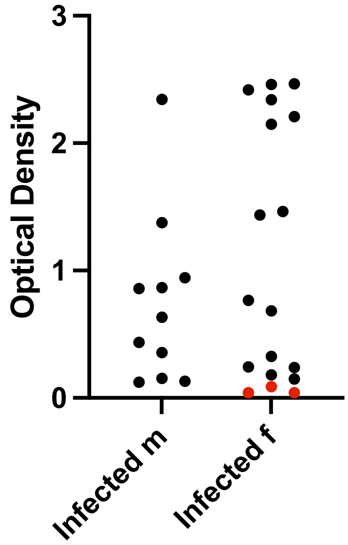

Figure S1. Results of the SARS-CoV-2 spike protein total antibody ELISA. Dots represent optical density (OD) of each sample. Cut-off value was OD<0,093. Sero-negative samples are represented as red dots.
