# Supplementary material for: Neuropsychiatric- and cognitive post-acute sequelae of SARS-CoV-2 infection – evidence from K18-hACE C57BL/6 J mice
Source: Int J Neuropsychopharmacol. 2025 Sep 30;28(10):pyaf072. doi: 10.1093/ijnp/pyaf072 (PMC12542986; doi:10.1093/ijnp/pyaf072)
Supplement: SupplFigureS2_310725_pyaf072 [file supplfigures2_310725_pyaf072.pdf]

Behaviour

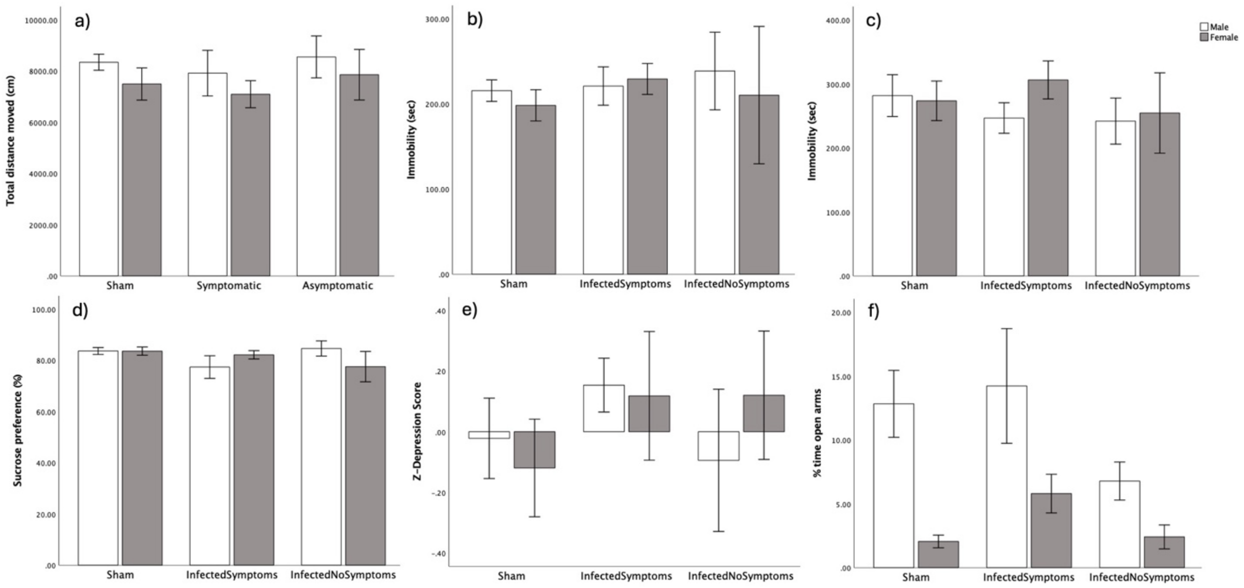

Figure S2. Behavioural results. a) Total distance moved in the OFT, b) Immobility time in the FST, c) Immobility time in the TST, d) Percent sucrose preference, e) Z – depression score, f) Percent time spend in the open arms of the EPM
