# Supplementary material for: Neuropsychiatric- and cognitive post-acute sequelae of SARS-CoV-2 infection – evidence from K18-hACE C57BL/6 J mice
Source: Int J Neuropsychopharmacol. 2025 Sep 30;28(10):pyaf072. doi: 10.1093/ijnp/pyaf072 (PMC12542986; doi:10.1093/ijnp/pyaf072)
Supplement: SupplFigureS3_310725_pyaf072 [file supplfigures3_310725_pyaf072.pdf]

Kynurenine pathway metabolite correlations with cognition

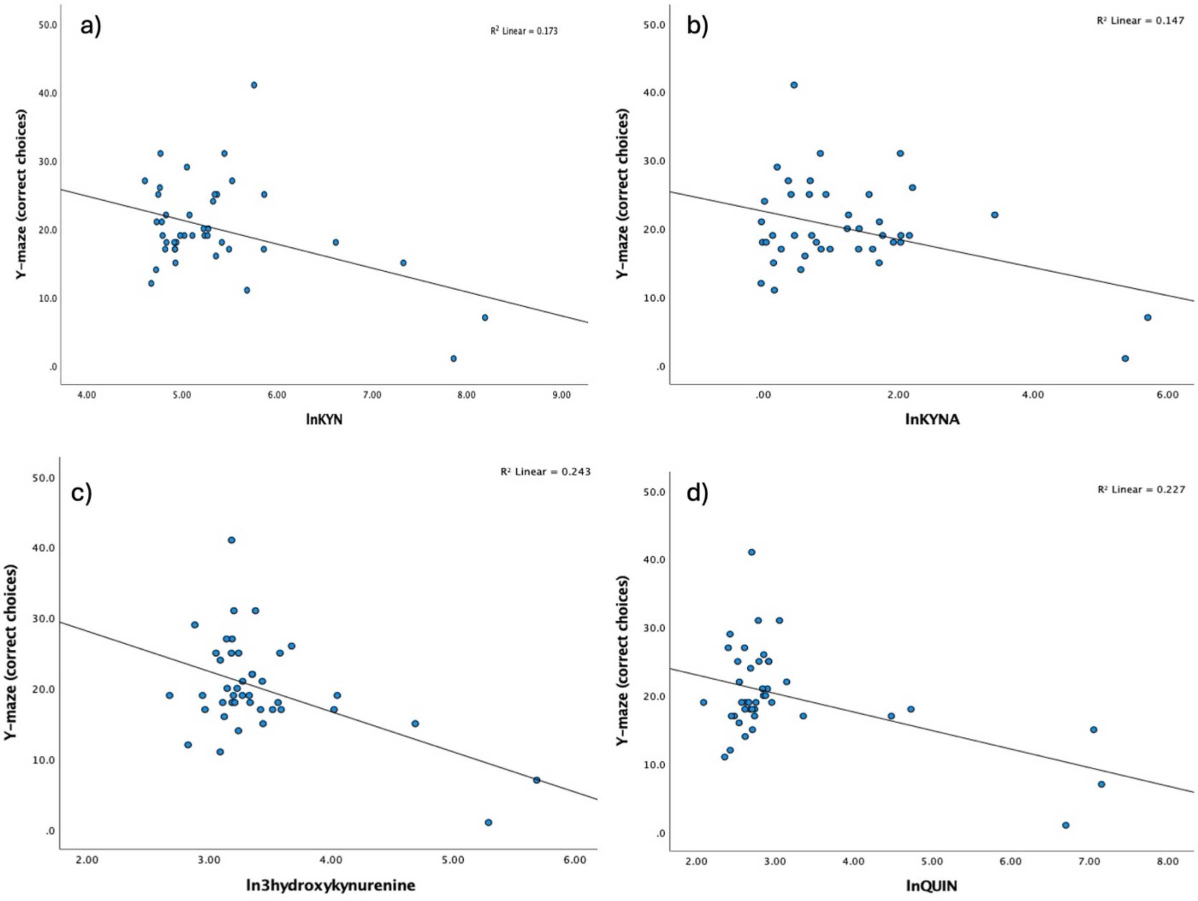

Figure S3. KP metabolites correlate negatively with Y-maze performance (Pearson Correlation). a) KYN (p< 0.001), b) KYNA (p<0.001), c) 3-HK (p<0.001), d) QUIN (p<0.01)
