# Supplementary material for: Neuropsychiatric- and cognitive post-acute sequelae of SARS-CoV-2 infection – evidence from K18-hACE C57BL/6 J mice
Source: Int J Neuropsychopharmacol. 2025 Sep 30;28(10):pyaf072. doi: 10.1093/ijnp/pyaf072 (PMC12542986; doi:10.1093/ijnp/pyaf072)
Supplement: SupplFigureS4_310725_pyaf072 [file supplfigures4_310725_pyaf072.pdf]

Microbiome

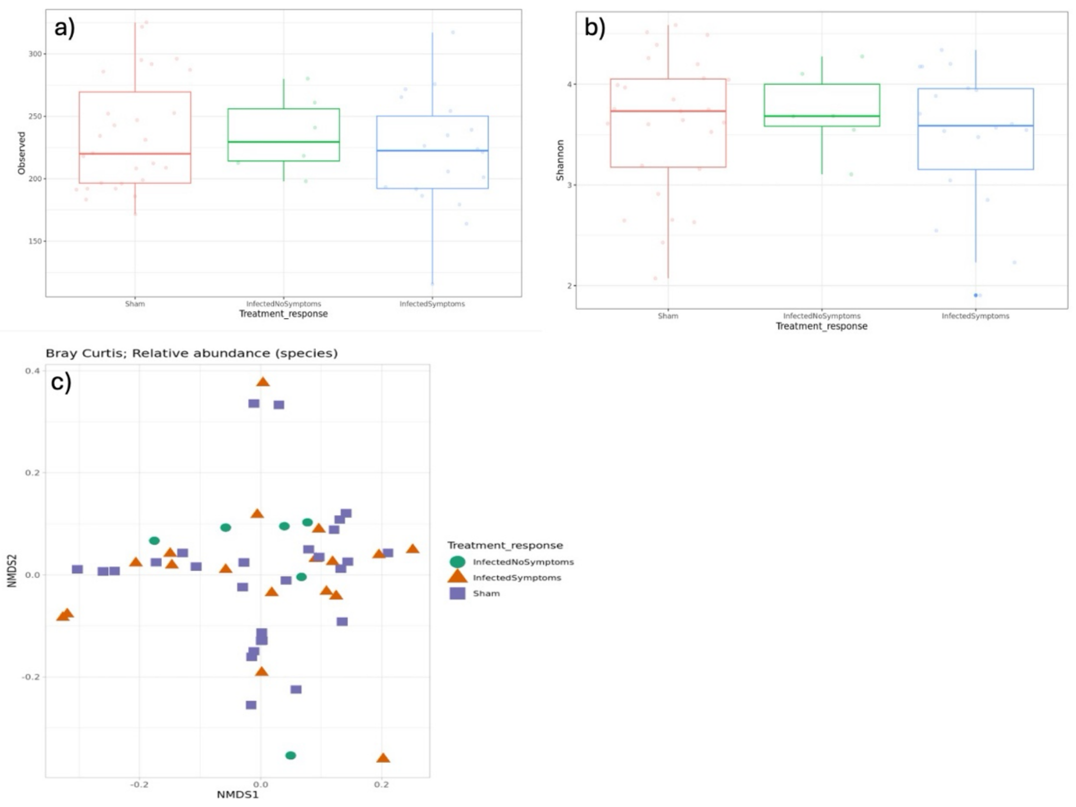

Figure S4. Illustration of the diversity levels in each sample based on infection response. Alpha diversity is illustrated in a) and b), beta diversity in c). a) illustrates observed species (richness), b) Shannon Index, and c) ordination plots using Bray-Curtis and a NMDS ordination method for species level taxonomic profiles
