# Supplementary material for: Neuropsychiatric- and cognitive post-acute sequelae of SARS-CoV-2 infection – evidence from K18-hACE C57BL/6 J mice
Source: Int J Neuropsychopharmacol. 2025 Sep 30;28(10):pyaf072. doi: 10.1093/ijnp/pyaf072 (PMC12542986; doi:10.1093/ijnp/pyaf072)
Supplement: SupplTableS1_310725_pyaf072 [file suppltables1_310725_pyaf072.pdf]

Cytokines, chemokines, and adhesion molecules

	Sham		Asymptomatic		Symptomatic		P-values	
	m	f	m	f	m	f	Main	Interaction
BLC	230±28	265±36	162±17	338±159	373±61	387±71	.01	.50
C5/C5a	324±54	277±37	208±22	271±51	283±32	297±34	.75	.61
CCL1	223±30	206±23	158±29	245±55	225±28	205±17	.97	.27
CCL11	190±23	163±15	135±14	182±9	190±24	162±12	.93	.28
CCL12	267±37	286±66	168±44	254±26	292±40	242±31	.75	.28
CCL2	207±24	202±22	135±17	217±52	258±34	202±21	.48	.12
CD54	782±84	2275±1352	684±61	912±152	8279±4939	3691±1316	<.01	.91
G-CSF	207±22	196±21	144±16	208±19	205±25	199±18	.81	.33
GM-CSF	286±25	251±19	226±24	306±21	319±40	270±24	.85	.16
I-TAC	274±40	370±139	187±45	321±112	247±32	219±21	.61	.39
IFN-G	324±55	323±44	214±26	304±31	309±22	279±26	.84	.45
IL-10	179±21	151±15	114±16	175±3	202±28	172±27	.61	.19
IL-12P70	128±15	172±48	81±13	119±18	141±11	123±14	.47	.41
IL-13	227±34	224±34	134±13	217±44	199±23	406±212	.70	.56
IL-16	434±67	459±80	245±43	363±61	534±86	411±41	.30	.40
IL-17	406±66	376±43	233±43	347±22	425±41	311±35	.49	.10
IL-1a	1275±181	1148±135	807±119	1068±133	1266±109	1026±83	.60	.32
IL-1B	210±31	192±21	113±14	200±29	227±32	166±15	.50	.06
IL-1ra	301±69	242±36	141±22	188±21	387±82	229±16	.07	.24
IL-2	281±65	265±38	127±19	187±23	263±47	205±22	.22	.34
IL-23	478±116	348±44	221±42	290±30	408±87	324±27	.31	.49
IL-27	767±180	688±106	321±73	396±7	789±146	502±59	.10	.28
IL-3	192±23	190±25	142±17	248±79	198±26	198±18	.93	.33
IL-4	274±36	322±72	214±38	343±99	252±23	291±31	.99	.64
IL-5	90±11	83±10	62±11	89±11	91±9	95±12	.72	.42
IL-6	140±17	126±12	102±11	131±9	145±13	150±24	.71	.53
IL-7	270±29	237±27	174±16	257±29	266±42	296±65	.82	.40
IP-10	314±44	275±31	169±34	271±33	464±119	272±27	.20	.12
KC	234±26	210±27	150±27	257±35	228±26	192±17	.84	.07
M-CSF	613±63	542±58	439±80	580±19	746±99	552±45	.50	.19
MIG	197±25	297±123	116±11	406±243	503±218	274±52	.24	.25
MIP-1a	224±33	278±85	117±31	287±112	239±27	185±20	.67	.07
MIP-1B	369±45	366±57	217±25	367±104	359±37	385±99	.63	.41
MIP-2	246±38	212±28	115±24	180±14	227±25	326±155	.38	.48
RANTES	228±43	181±24	99±17	150±15	419±132	223±31	0.01	.28
SDF-1	2486±549	2076±305	1277±281	1713±191	2403±583	1809±161	.53	.56
TARC	269±33	244±20	206±39	255±14	285±46	224±19	.85	.35
TIMP-1	410±46	370±33	296±61	942±364	497±88	357±31	.42	.01
TNF-a	1103±141	918±99	714±40	1015±138	1060±146	985±83	.78	.29
TREM-1	301±46	189±22	116±17	278±15	237±55	184±19	.59	.01

Table S1. Mean relative values ±SEM of the measured cytokines, chemokines and adhesion molecules. Main effect of infection response and interaction between infection response and sex is presented with p-values from the 2-way ANOVA.
