# Supplementary material for: Neuropsychiatric- and cognitive post-acute sequelae of SARS-CoV-2 infection – evidence from K18-hACE C57BL/6 J mice
Source: Int J Neuropsychopharmacol. 2025 Sep 30;28(10):pyaf072. doi: 10.1093/ijnp/pyaf072 (PMC12542986; doi:10.1093/ijnp/pyaf072)
Supplement: SupplTableS2_310725_pyaf072 [file suppltables2_310725_pyaf072.pdf]

Monoamine neurotransmitters and-metabolites

	Sham		Asymptomatic		Symptomatic		P-values	
	m	f	m	f	m	f	Main	Interaction
NA	.93±.09	1.14±.09	1.20±.09	1.34±.2	1.02±.1	1.1±.06	.33	.72
DA	.11±.03	2.67±2.5	.070±.002	.093±.01	.093±.01	.21±.1	.73	.73
DOPAC	.031±.003	.55±.5	.035±.01	.040±.004	.046±.02	.080±.02	.74	.72
DOPAC/DA	.34±.024	.53±.066	.50±.15	.43±.018	.44±.11	.58±.11	.62	.61
HVA	.0012±.0001	.0014±.0003	.002±.0006	.0023±.0008	.0012±.0002	.0018±.0003	.14	.76
5-HT	1.01±.1	1.18±.09	1.09±.05	1.63±.2	1.05±.2	1.03±.2	.37	.44
5-HIAA	.25±.02	.62±.08	.24±.009	.53±.1	.43±.1	.64±.1	.46	.70
5-HIAA/5-HT	.26±.014	.54±.056	.22±.0014	.32±.038	.37±.084	.62±.065	.05	.63

Table S2. Mean values ±SEM of noradrenalin (NA), dopamine (DA), 3,4-dihydroxyphenyl-acetic acid (DOPAC), homovanilic acid (HVA), 5-hydroxytryptamine (5-HT), and 5-hydroxyindoleacetic acid (5-HIAA) in hippocampus of the mice. All values are reported as nmol/g wet weight of tissue. Main effect of infection response and interaction between infection response and sex is presented with p-values from the 2-way ANOVA.
